# The influence of *SnoN* gene silencing by siRNA on the cell proliferation and apoptosis of human pancreatic cancer cells

**DOI:** 10.1186/s13000-015-0267-3

**Published:** 2015-04-18

**Authors:** Chengli Liu, Hui Zhang, Xiaoxia Zang, Cheng Wang, Yalin Kong, Hongyi Zhang

**Affiliations:** Department of Hepatobiliary Surgery, Air Force General Hospital of PLA, 30 Fucheng Road, Beijing, 100142 China; Department of Stomatology, Air Force General Hospital of PLA, Beijing, China

**Keywords:** *SnoN* gene, Proliferation, Apoptosis, Pancreatic cancer (PC)

## Abstract

**Background:**

The prognosis for pancreatic cancer (PC) is very poor. The *SnoN* gene may have a role in cell proliferation and apoptosis in human cancer. However, the influence of *SnoN* on cell proliferation and apoptosis in human PC cells remains unknown.

**Methods:**

*SnoN* expression was assessed in SW1990 PC cell lines using real-time polymerase chain reaction (PCR). A luciferase reporter assay was used to confirm the target associations. The effect of *SnoN* on cell proliferation *in vitro* was confirmed using Cell Counting Kit-8. Apoptosis was confirmed using flow cytometry. Gene and protein expression were examined using real time PCR and Western blotting, respectively.

**Results:**

*SnoN* siRNA significantly inhibited the growth of SW1990 cells by decreasing cell proliferation (*P* < 0.05) and increasing cell apoptosis (*P* < 0.05), compared with the blank group and the negative control group. The highest inhibition of cell proliferation appeared at 3 days post-transfection. Cell apoptosis more obvious at 48 h after transfection.

**Conclusions:**

In summary, our results reveal that the RNAi-mediated downregulation of *SnoN* effectively inhibited the proliferation of PC cells. *SnoN*-siRNA also enhanced SW1990 PC cell apoptosis. These findings indicate that *SnoN* gene plays an important role in pancreatic cancer development, and might serve as a potential therapeutic target for pancreatic cancer. However, further *in vivo* studies are needed to clarify the influence of *SnoN* gene silencing by siRNA on pancreatic cancer therapy.

**Virtual slides:**

The virtual slide(s) for this article can be found here: http://www.diagnosticpathology.diagnomx.eu/vs/7609324661510147

## Background

Pancreatic cancer (PC) is one of the most fatal malignant diseases worldwide. The incidence of PC is lower than that of many other types of cancer. However, it is the fourth most common cause of death from cancer [1]. Because of nonspecific incipient symptoms and early metastasis, PC is highly malignant and invasive, resulting in poor prognosis [2]. Previous statistics for 2002–2008 from the US National Cancer Institute showed an overall 5-year relative survival rate of 5.8% and a one-year mortality rate of 90%, with a median survival of less than 6 months [3,4].

A number of studies [5-8] have showed that the development and progression of PC are linked with complex gene regulation, such as the inactivation of tumour suppressor genes, the activation of proto-oncogenes, abnormal regulation of cell proliferation and apoptosis adjustment disorders, and abnormal expression of growth factors and their receptors. In recent years, although targeted therapies in PC treatment have been gradually developed, the PC survival rate has not improved. Therefore, there is an urgent need for the identification of possible associated factors and novel therapeutic targets for PC.

The *SnoN* gene, a member of the Ski family of proto-oncogenes that was originally identified based on sequence homology with v-Ski, encodes an oncoprotein that can induce anchorage-independent growth of chicken and quail embryo fibroblasts when overexpressed [9-12]. *SnoN* is highly expressed in human cancer cells of the oesophagus, lung, vulva, stomach, ovary, pancreas, and breast [10,13-15]. *SnoN* overexpression in human cancer cells may result in gene amplification, transcriptional activation, and increased protein stability [14,16-19]. Moreover, *SnoN* was recently shown to silence the alpha fetoprotein gene, cooperating with p53 to negatively regulate transforming growth factor beta (TGF-β) signalling [19-21]. Although several previous studies investigated the effects of *SnoN* on the cell proliferation and apoptosis of ovarian, lung, breast, oesophageal, and colon cancers [22-26], the role *SnoN* plays in pancreatic cancer remain unknown.

In the present study, we established a pancreatic cancer cell line that stably silenced the *SnoN* gene by siRNA, and investigated the effects of *SnoN* on the proliferation and apoptosis of pancreatic cancer cells *in vitro*. This study may provide experimental evidence for gene therapy in PC.

## Methods

### Ethical approval

This study was approved by the Ethics Committee of Air Force General Hospital of PLA.

### Cell culture

The SW1990 human pancreatic cancer cells were obtained from the Cell Bank of the Chinese Academy of Sciences (Shanghai, China) and cultured in Dulbecco’s modified Eagle’s medium (DMEM, HyClone, Logan, UT, USA) supplemented with 10% foetal bovine serum (FBS, HyClone, Logan, UT, USA), 100 U/mL penicillin and 100 μg/mL streptomycin. Cells were maintained in a 37°C humidified incubator containing 95% air and 5% CO_2_.

### Reagents

Opti-MEM medium and Lipofectamine 2000 (Lipo) were obtained from the Gibco-Invitrogen Corporation (USA). Trypsin was obtained from the HyClone Corporation (USA). The β-actin mAb was purchased from the Shanghai Kangcheng Biotechnology Corporation (Shanghai, China). The *SnoN* mAb was purchased from the Abcam Corporation (UK). The cDNA synthesis kit and TRIzol kit were purchased from Bao Biotechnology Corporation (Dalian, China). The RNA PCR kit was obtained from TaKaRa Corporation (Japan). The Western Blotting Detection system was obtained from Thermo Corporation (USA). The cell lysate and bicinchoninic acid (BCA) protein assay kit was purchased from Shanghai Biyuntian Biotechnology Corporation (Shanghai, China). The Cell Counting Kit-8 was obtained from Dojindo Chemical Corporation (CCK-8, Japan). The Annexin V FITC/PI assay kit was purchased from Invitrogen Corporation (USA).

### ***SnoN*** siRNA sequences and PCR primers

*SnoN* siRNAs, labelled by florescence FAM (FAM-siRNAs), and negative control siRNAs were synthesised by Shanghai Jima Biotechnology Co., Ltd. (Shanghai, China). For the *SnoN* siRNA-A, the sense strand was 5’-GGGCUUUGAAUCAGCUAAATT-3’ and the antisense strand was 5’-UUUAGCUGAUUCAAAGCCCTT-3’. For the *SnoN* siRNA-B, the sense strand was 5’-GGCCCAGUUAAAGGAAACUTT-3’ and the antisense strand was 5’-AGUUUCCUUUAACUGGGCCTT-3’. For the *SnoN* siRNA-C, the sense strand was 5’-GAGGCAAGUAAGUCCAUAUTT-4’ and the antisense strand was 5’-AUAUGGACUUACUUGCCUCTT-3’. For the negative control siRNA, the sense strand was 5’-UUCUCCGAACGUGUCACGUTT-3’ and the antisense strand was 5’-ACGUGACACGUUCGGAGAATT-3’. The *SnoN* primers were forward, 5’-AGAGACTCTGTTTGCCCCAAGT-3’ and reverse, 5’-CATGCTAAACTTCTCCTTCATTTC-3’. The β-actin primers were forward, 5’-TTCTGTGGCATCCACGAAACT-3’ and reverse, 5’-GAAGCATTTGCGGTGGACGAT-3’.

### siRNA transfection

The pancreatic cancer cells were seeded at 1×10^5^ cells/well in 24-well plates 1 day before transfection. Medium without antibiotics was added to each well so that the cells grew to 50-70% confluence, when the transfection was conducted. The siRNA-Lipo mixture was prepared according to the manufacturer's instructions. To test the transfection efficiency of the FAM-siRNAs-Lipo mixture at different concentrations, 0, 1, 1.5 and 2 μl of Lipo were diluted with 50 μl Opti-MEM, and 0, 10, 15 and 20 μl FAM-siRNAs, respectively, were added at 50 μl/well and mixed. The 100 μl mixture was added to 300 μl of Opti-MEM including the pancreatic cancer cells. The concentrations of the four groups were 0 nmol/L, 50 nmol/L, 75 nmol/L, and 100 nmol/L, respectively. Six hours later, the medium was replaced with DMEM supplemented with 10% FBS. The expression of FAM-siRNAs was analysed with a flow cytometer.

### Quantitative RT-PCR

Total RNA was extracted from cells using TRIzol reagent and reverse transcribed to cDNA using a first-strand cDNA synthesis kit according to the manufacturer’s instructions. The efficiency of the *SnoN* siRNAs was screened using RT-PCR and real-time PCR. Real-time PCR was conducted on the Exicycler™ 96 florescence quantitative instrument (Bioneer, Daejeon, Korea). PCR conditions and analysis were consistent with previously published methods [27].

### Western blotting

*SnoN* protein expression was measured by Western blotting. β-actin was used as an internal control. Cells were lysed using cell lysis buffer, and the protein concentrations were quantified using the BCA assay method. Cellular proteins were dissolved in sample loading buffer and run on 7.5% sodium dodecyl sulphate-polyacrylamide gel electrophoresis (SDS-PAGE) gels (100 V, constant voltage, 60 min). *SnoN* protein was electrotransferred onto PVDF membranes (4°C, 280 mA, 60 min). The membranes were rinsed with PBS and blocked with 10% non-fat milk in PBS for 2 h at room temperature. Primary antibody was used at the following dilutions. After primary antibody incubation, membranes were rinsed in TBS-T wash buffer 3 times for 10 min each. Then, secondary antibody (1: 2,000) was incubated for 2 h at room temperature and rinsed in TBS-T wash buffer 3 times for 10 min each. Developed films were digitised by scanning, and the optical densities were analysed with the image software.

### Cell proliferation analysis

Cell viability was measured using the CCK-8 assay. Cells were seeded into 96-well plates at a density of 3 × 10^3^ cells/well and placed in an incubator until the cells grew to confluence. Subsequently, 100 μl of CCK-8 solution was added at different time points (0, 24, 48, 72, and 96 h), and the samples were incubated at 37°C for 1.5 h. The absorbance value of each well was measured at 450 nm using a microplate reader. Non-transfected cells were set as the blank control group. Cells treated with negative siRNA transfection were set as the negative control group.

### Analysis of cell apoptosis

An annexin V FITC/PI assay kit was used to detect cell apoptosis. Cells were divided into three groups (blank control group, negative control group, and transfection group). At 50% - 70% confluence, the cells were trypsinised and collected. After washing with PBS, 500 μl of annexin-binding buffer was added to resuspend the cells. After the addition of 5 μl Alexa-labelled annexin V and 1 μl PI, the cells were incubated at room temperature in the dark for 15 min and then detected by flow cytometry.

### Statistical analysis

Data are presented as the mean ± standard deviation and were analysed using one-way analysis of variance (ANOVA) between groups using the SPSS 16.0 software. The differences among multiple mean values were evaluated using ANOVA. The differences between two mean values were estimated using an independent-samples *t*-test. The differences among the groups were analysed using the *χ*2 test. A *P* < 0.05 was considered statistically significant.

## Results

### Transfection efficiency

The transfection efficiency was measured by flow cytometry. When the concentrations of the FAM-siRNA mixture were 0 nmol/L, 50 nmol/L, 75 nmol/L, and 100 nmol/L, the transfection efficiencies were 9.38%, 29.9%, 57.4%, and 87.5%, respectively. We found that the optimal transfection concentration was 100 nmol/L.

### Silencing efficiency of ***SnoN*** siRNAs

**Figure 1:**
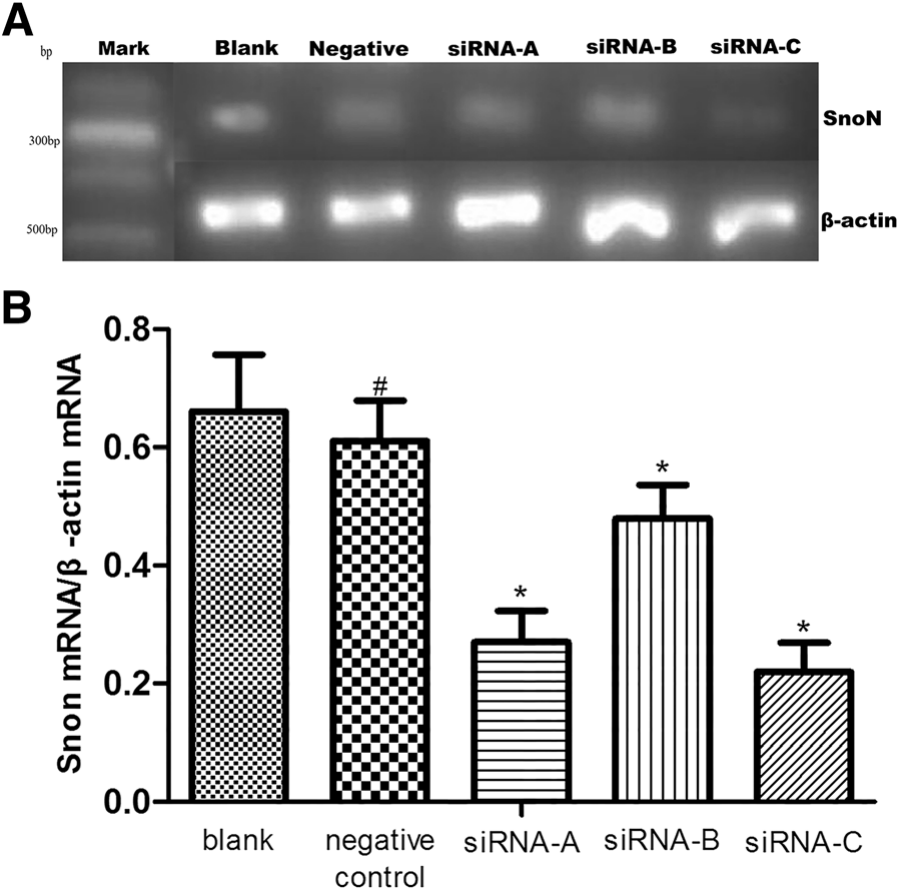
Expression of *SnoN* mRNA in SW1990 cells. **A** The effect of three different siRNA sequences on silencing the expression of *SnoN* mRNA in SW1990 cells. **B** Real-time PCR was used to detect the *SnoN* mRNA expression level. ^#^P >0.05 and ^*^P < 0.05, compared with the blank control group.

Three *SnoN* siRNA sequences were used to determine the silencing efficiency on *SnoN* mRNA expression in pancreatic cancer cells. The results after real-time PCR analysis showed little difference in band brightness between the five β-actin groups, which revealed that the RNA template for reverse transcription was equal in each group. No significant difference was observed between the blank and negative control groups. However, lower band brightness was evident in the three siRNA groups, compared with the blank or negative control groups (P < 0.05). According to the ratio of the optical density values between the *SnoN* and β-actin bands, similar results were observed. The *SnoN* siRNA-C was found to have the most powerful silencing effect on the *SnoN* gene (Figure 1).

### Screening efficiency of ***SnoN*** protein expression using Western blotting

**Figure 2:**
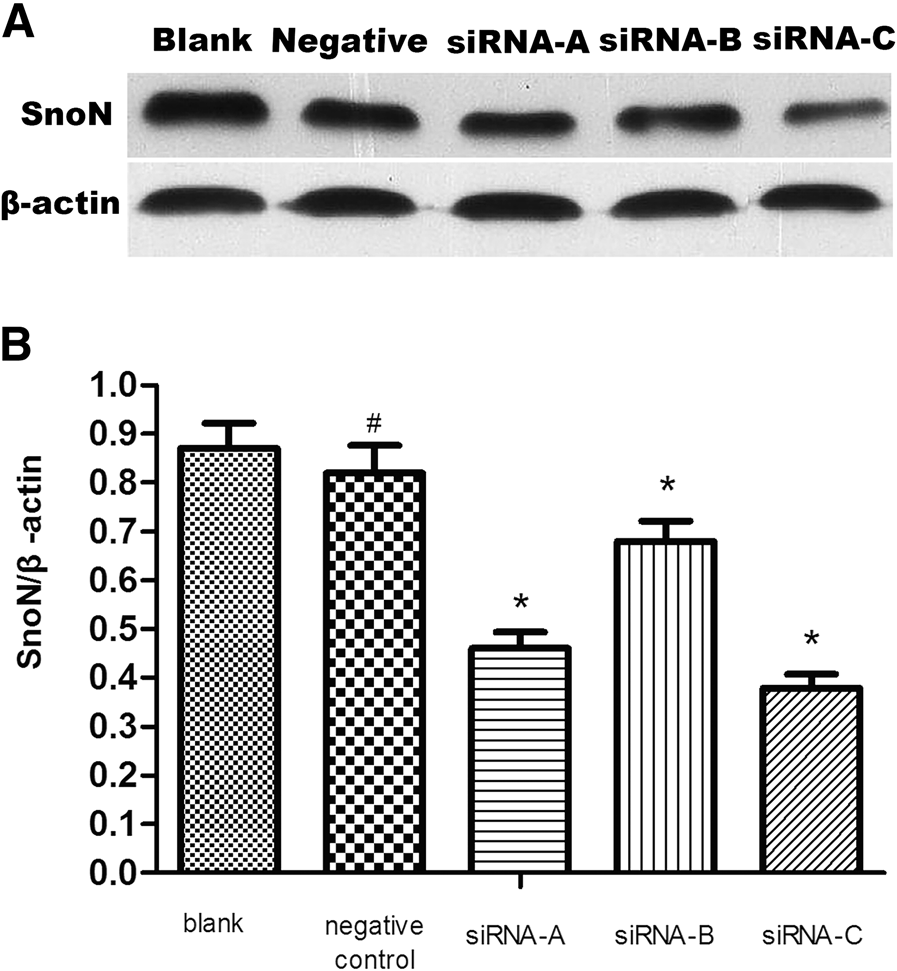
Expression of *SnoN* protein in transfected SW1990 cells. **A** Western blotting was used to detect the cyclin *SnoN* protein expression level in each group. Representative results obtained from three repeated experiments are presented in the figure. **B** β-actin was used as an internal reference to analyze the relative protein expression level of cyclin *SnoN*, n = 3. ^#^P >0.05 and ^*^P < 0.05, compared with the blank control group.

The pooled results demonstrated that protein expression decreased in the three siRNA groups compared with the blank or negative control groups (P < 0.05). *SnoN* siRNA-C had the most powerful inhibitory effect on *SnoN* protein expression (Figure 2).

### The effect of silencing ***SnoN*** on cell proliferation

**Figure 3:**
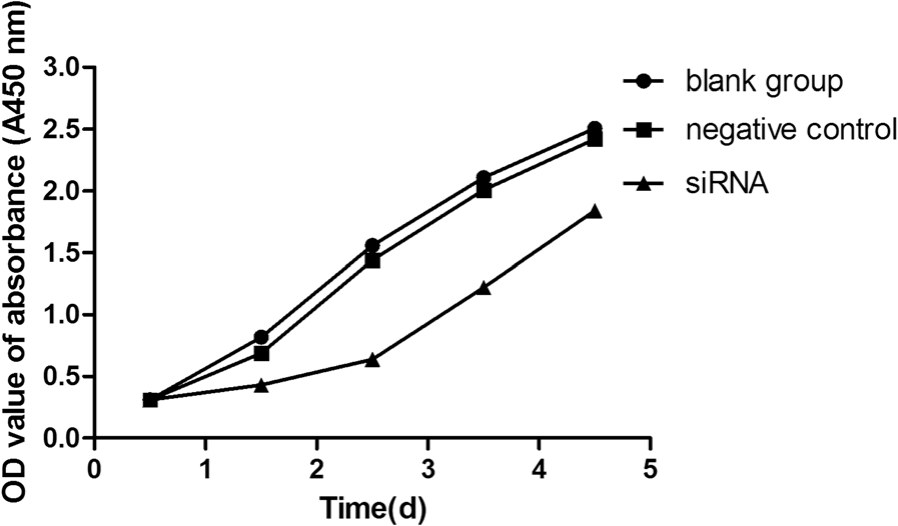
The CCK-8 method was used to detect cell proliferation. Cells were seeded in 96-well plates, and the absorbance of each well was detected at different time points at 450 nm, n = 6.

The growth curve representing cell proliferation was drawn according to the results of the CCK-8 assay. The *SnoN* siRNA-C group was significantly decreased at each day after transfection (P < 0.05). The lowest cell viability was observed at 3d after transfection, and the recovery of cell viability started at 4d after transfection (Figure 3).

### The effect of silencing ***SnoN*** on cell apoptosis

**Figure 4:**
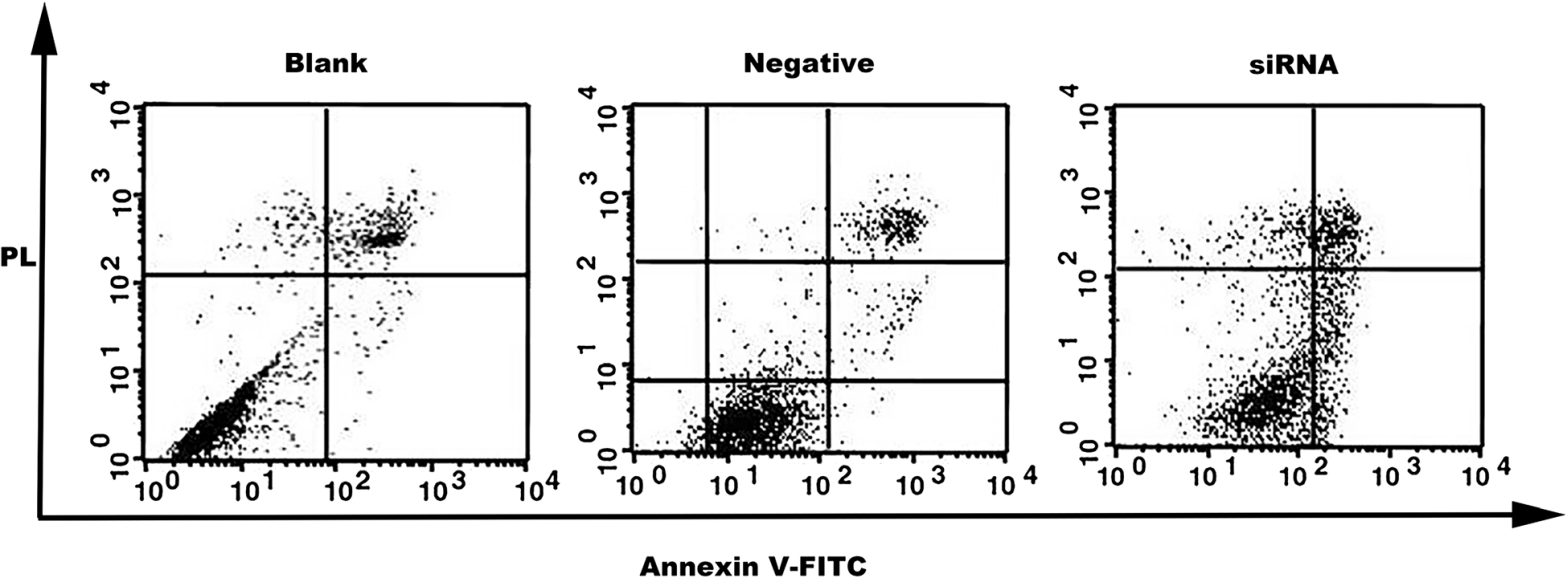
The effect of SnoN siRNA-C on apoptosis of SW1990 cells was tested by FCM.

The apoptotic cells increased after pancreatic cancer cells were transfected with *SnoN* siRNA-C (P < 0.05). This was more obvious at 48 h after transfection. There was a significant difference between the *SnoN* siRNA group and the negative control group (P < 0.05). There was a significant difference between the *SnoN* siRNA group and the blank control group (P < 0.05). Moreover, there were no significant differences between the negative control group (negative siRNA transfected cells) and the blank control group (non-transfected cells) (Figure 4, Table 1).

**Table 1:** **The effect of** ***SnoN*** **-siRNA on apoptosis of PC cells (n = 3)**

**Group**	**Time (d)**	**Apoptosis (%)**
Blank control	24	0.26 ± 0.27
	48	0.67 ± 1.38
	72	1.77 ± 1.54
Negative control	24	0.29 ± 0.31
	48	0.72 ± 1.15
	72	2.13 ± 1.97
*SnoN*-siRNA-C	24	0.56 ± 0.29^*^
	48	6.94 ± 2.17^*^
	72	7.45 ± 2.01^*^

^*^P < 0.05 vs. blank control.

## Discussion

The present work provides evidence of the importance of *SnoN* for cell growth in MPM. The transient *SnoN*-silencing caused a decrease in the proliferation rate of the pancreatic cancer cell line and an increase in the apoptosis rate.

Fewer than 5% of patients with PC achieve 5-year survival, which reveals the poor prognosis of this cancer [28]. Therefore, to improve modern cancer therapy, numerous scholars have an ongoing interest in the identification of signalling pathways and genes that might play key roles in carcinogenesis and the development of resistance to anti-tumour drugs, with the hope of identifying putative biomarkers and/or therapeutic targets [29,30]. Applying molecular approaches, a large set of genes has been investigated for overexpression in pancreatic cancer [31-33]. However, more genes that may have an effect in the progression of PC need to be investigated.

The human *SnoN* gene is located at chromosome 3q26.2. *SnoN* is widely expressed in adult and embryonic cells. *SnoN* expression can be regulated at the level of gene amplification, transcriptional activation and protein stability. High expression of *SnoN* is found in many human cancers [22-26]. However, not all studies were consistent with upregulated *SnoN* expression in human cancers. While some studies reported an increase in *SnoN* RNA and protein in some cancer tissues and noted that this higher *SnoN* expression correlated with poor differentiation, deeper invasion and poor patient survival [14,15,25], others detected a decrease in *SnoN* expression in similar cancers, particularly in dysplastic and highly invasive cancers [34-36]. It appears that *SnoN* can also act as a negative regulator of tumour progression. In a recent study [21], *SnoN* was observed to cooperate with p53 in the silencing of the alpha fetoprotein gene, which is aberrantly overexpressed in liver cancer cells.

Moreover, *SnoN* and its structurally and functionally related protein Ski are negative regulators of TGF-β-induced transcription [37]. The level of *SnoN* is directly linked to its ability to repress TGF-β signalling, but this activity may be cell-type specific [38]. *SnoN* has been reported to be both a tumour promoter and a tumour suppressor [39]. Over-expression of *SnoN* leads to resistance to TGF-β-induced growth arrest and formation of mammary tumours in cooperation with polyoma middle T-antigen [17]. Furthermore, previous studies have shown that *SnoN* interacts directly with Smad2, Smad3, and Smad4 and represses their ability to activate expression of TGF-β target genes by disrupting the formation of an active heteromeric Smad complex, recruiting a transcriptional corepressor complex, and by blocking the interaction of transcriptional coactivators with Smad2 and Smad3 [40,41]. A number of investigations have indicated that PC is closely associated with the TGF-β/Smad pathway, and Smad4 is inactivated in nearly 60% of PC [42]. However, the influence of *SnoN* gene expression on cell proliferation and apoptosis in human pancreatic cancer cells remains unclear.

Cell proliferation and apoptosis are important to the oncogenesis and chemotherapy resistance of pancreatic cancer cells [8]. In this study, we analysed the suppressive effect of PC cell proliferation and apoptosis by siRNA silencing of *SnoN* expression. The results showed that the proliferation of the *SnoN*-siRNA-transfected cells was slower than blank and negative group, suggesting a fundamental role for *SnoN* in the development of PC. Therefore, through silencing *SnoN* gene, development of PC might be alleviated. To date, this is the first study in which RNAi-mediated *SnoN* expression was shown to inhibit PC cell proliferation and induce apoptosis. This finding is consistent with the previous results that *SnoN* promotes tumour proliferation and induces apoptosis in other cancers [14,15,25]. However, because the exact effect of *SnoN* expression on human cancers remains unclear, and multiple signalling pathways are thought to make important contributions to PC progression, further studies are necessary to clarify these complex mechanisms. Moreover, our study has a limitation, in that we only proved that RNAi inhibited *SnoN* expression *in vitro*. Therefore, further *in vivo* studies are needed to verify the effect and risk of *SnoN* knockdown by *SnoN*-siRNAs for PC therapy.

## Conclusions

In summary, our results revealed that the RNAi-mediated downregulation of *SnoN* effectively inhibited the proliferation of PC cells. Meanwhile, *SnoN*-siRNA also enhanced PC cell apoptosis. These findings indicated that *SnoN* plays an important role in pancreatic cancer development, and might serve as a potential therapeutic target for pancreatic cancer. However, further *in vivo* studies are needed to clarify the influence of *SnoN* gene silencing by siRNA on pancreatic cancer therapy.
